# Supplementary material for: Bright Fluorescent Vacuolar Marker Lines Allow Vacuolar Tracing Across Multiple Tissues and Stress Conditions in Rice
Source: Int J Mol Sci. 2020 Jun 12;21(12):4203. doi: 10.3390/ijms21124203 (PMC7352260; doi:10.3390/ijms21124203)
Supplement: Supplementary file 1 [file ijms-21-04203-s001.zip › tableS1-2.pdf]

Table S1. The accession ID of Arabidopsis and rice TIPs

Gene	Locus tag
AtTIP1;1	AT2G36830
AtTIP1;2	AT3G26520
AtTIP1;3	AT4G01470
AtTIP2;1	AT5G47450.1
AtTIP2;2	AT4G17340.1
AtTIP2;3	AT5G47450
AtTIP3;1	AT1G73190
AtTIP3;2	AT1G17810
AtTIP4;1	AT2G25810
AtTIP5;1	AT3G47440
OsTIP1;1	LOC_Os03g05290
OsTIP1;2	LOC_Os01g74450
OsTIP2;1	LOC_Os02g44080
OsTIP2;2	LOC_Os06g22960
OsTIP3;1	LOC_Os10g35050
OsTIP3;2	LOC_Os04g44570
OsTIP4;1	LOC_Os05g14240
OsTIP4;2	LOC_Os01g13130
OsTIP4;3	LOC_Os01g13120
OsTIP5;1	LOC_Os04g46490

Table S2. The primers used in this study

Gene Name	Forward Primer	Reverse Primer
ZmUbi	ATGACCATGATTACGCAGTGCAGCGTGACCCGGT	TACCGAGCTCGAATTCGGATCCTCTAGAGTCGACCTGCAGA
eGFP	ATTCGAGCTCGGTACCATGGTGAGCAAGGGCGAGGA	CTAGAGGATCCCCGGGTACCCTTGTACAGCTCGTCCATGCC
mCherry	CGGGGATCCTCTAGAATGGTGAGCAAGGGCGAGGA	GCAGGTCGACTCTAGACTTGTACAGCTCGTCCAT
OsTIP1;1	GCAGGCATGCAAGCTTATGCCGATCCGCAACATCGC	ACCTGTAATTCACACGTGTTAGTAGTCGGTGGTGGGGA
AtVAMP711	AGTCTAGAGTCGACCTGCAGATGGCGATTCTGTAC	TACGAACGAAAGCTCTGCAGTTAAATGCAAGATGG
