# Supplementary material for: Bright Fluorescent Vacuolar Marker Lines Allow Vacuolar Tracing Across Multiple Tissues and Stress Conditions in Rice
Source: Int J Mol Sci. 2020 Jun 12;21(12):4203. doi: 10.3390/ijms21124203 (PMC7352260; doi:10.3390/ijms21124203)
Supplement: Supplementary file 1 [file ijms-21-04203-s001.zip › ijms-789419-supplementary.docx]

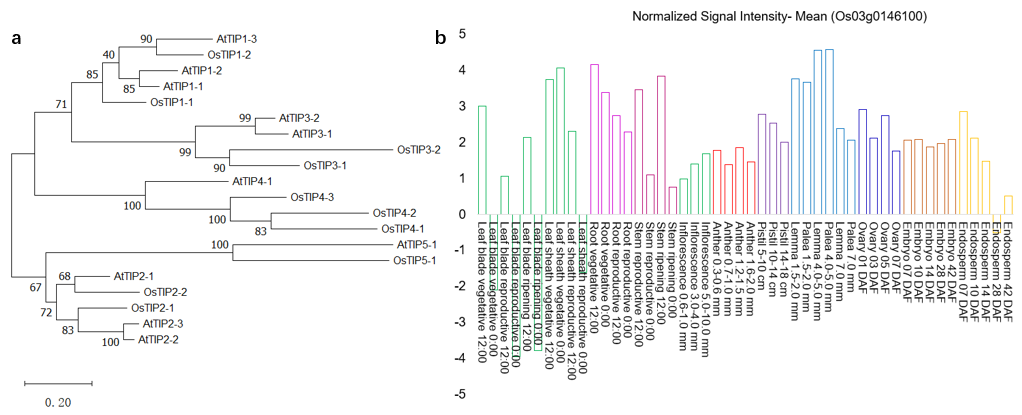


**Figure 1.** Informatics data on OsTIP1;1. (**a**) A homology tree of rice and Arabidopsis TIPs (tonoplast intrinsic proteins). The tree was made by MEGA (Molecular Evolutionary Genetics Analysis) program with the full protein sequence downloaded from NCBI (National Center for Biotechnology Information). Those numbers above the evolution tree represent the reliability, and the branch lengths of tree were measured in the number of substitutions per site. Accession IDs are indicated in Table S1. Scale bars represent the substitution rates. (**b**) Spatio-temporal expression pattern of *OsTIP1;1* downloaded from RicEXpro (http://ricexpro.dna.affrc.go.jp/).


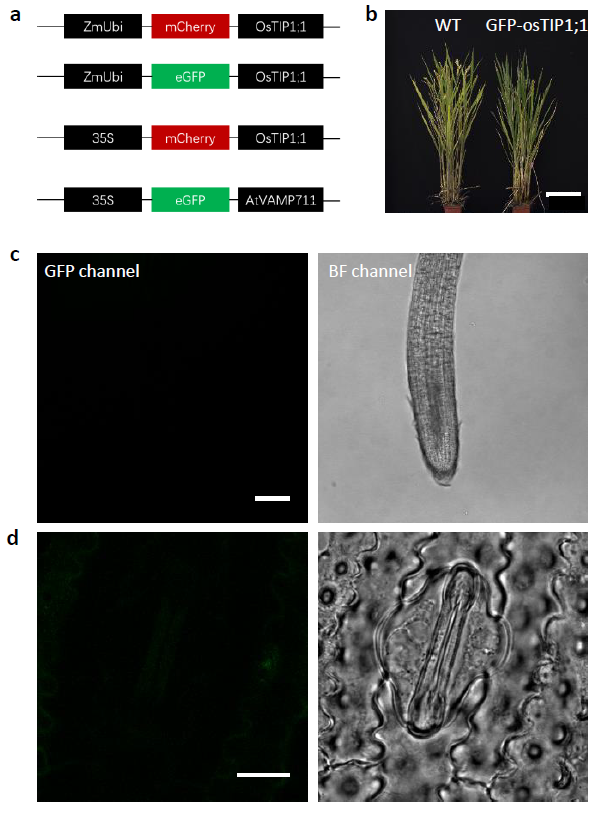


**Figure 2.** The transgenic plant phenotype of GFP-OsTIP1;1 and the auto-fluorescence control in wild type. (**a**) Schematic design diagram of three constructs used in this study. The maize ubiquitin promoter (ZmUbi) driven vectors were transformed into rice to obtain stable transformed plants. The 35s promoter driven vectors were used for transient expression in tobacco or rice protoplast. (**b**) The transgenic rice plants have no obvious developmental difference compared to wild type (WT). The scale bar: 20 cm. (**c**) The images of the WT root under the same settings to imaging GFP-OsTIP1;1. (**d**) The images of the WT stomata under the same settings to imaging GFP-OsTIP1;1. Note that no signal or super weak signal was seen in wild type. Scale bars: 10 µm.


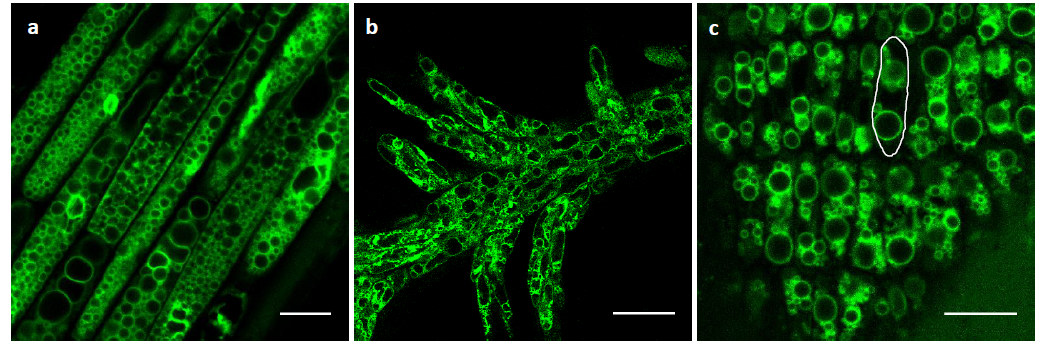


**Figure 3.** Vacuole morphology varies in different reproductive organs. Vacuoles in anther filament ((**a**), Scale bar: 10 µm), pistil ((**b**), Scale bar: 50 µm), and ovule ((**c)**, Scale bar: 10 µm) epidermis vary substantially in size and organization. The white line-enclosed area in (**c**) indicates the cell edge.


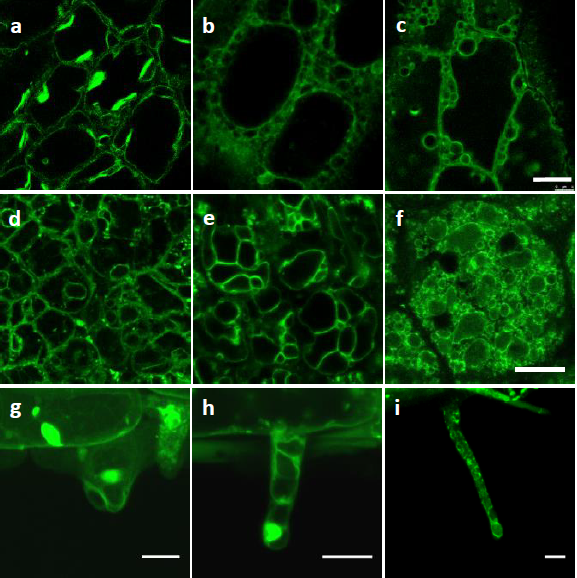


**Figure 4.** Vacuole morphology changes during development in rice anthers, endosperm and root hairs. (**a**–**c**) Vacuole changes in anther epidermis during anther growth. (**a–c**) Correspond to pollen developmental stages 8, 9, and 11, respectively. (**d–f**) Vacuole progression in developing endosperm. (**d**) Depicts endosperm a few days after pollination. (**f**) Shows the storage vacuoles in drying seeds. The age of seeds in (**e**) was between that of (**d**) and (**f**). (**g–i**) Vacuole morphology in developing root hairs. Images are max-projections of z-stacks. Scale bars: 10 µm.


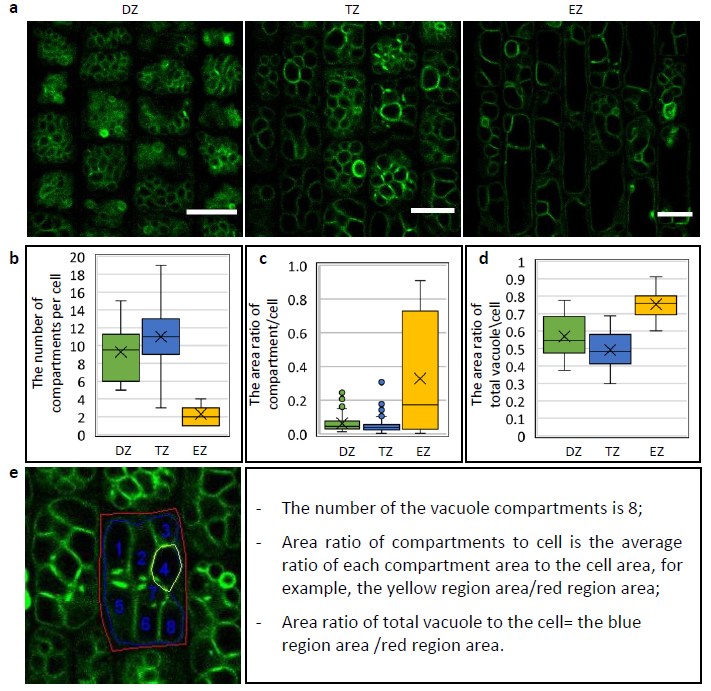


**Figure 5.** The compartmentalized vacuoles in rice radical root cells. (**a**) The single images of GFP–OsTIP1;1 in the epidermal cells of radical root. Approximate sub-apical root regions are indicated as elongation zone (EZ), transition zone (TZ), and dividing zone (DZ). (**b**) The vacuole compartment numbers per cell. (**c**) The area ratio of compartment to the cell. (**d**) The area ratio of total vacuole to the cell. (**e**) The explanation for the measurements in (**b)** to (**d**). Scale bars: 10 µm.


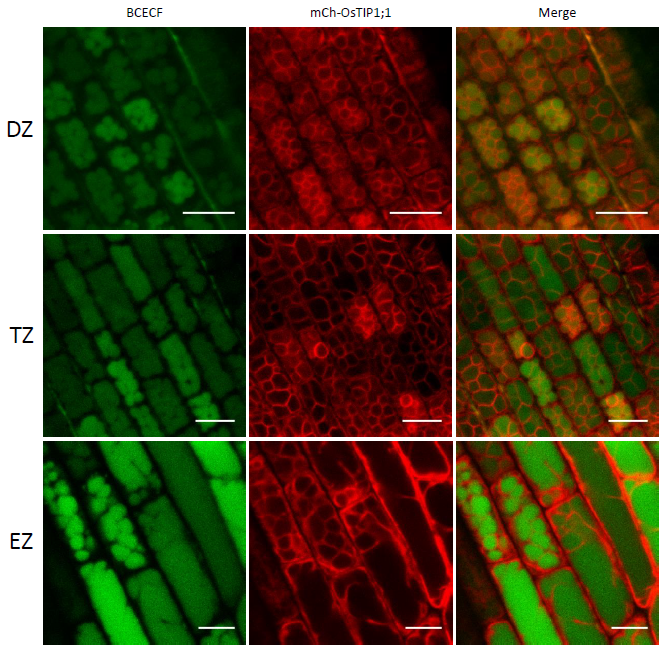


**Figure 6.** Relative pH estimates of the vacuole lumen. The vacuole lumens stained by BCECF were surrounded by the tonoplasts labelled by mCh-OsTIP1;1 in different root zones. Scale bars: 10 µm.


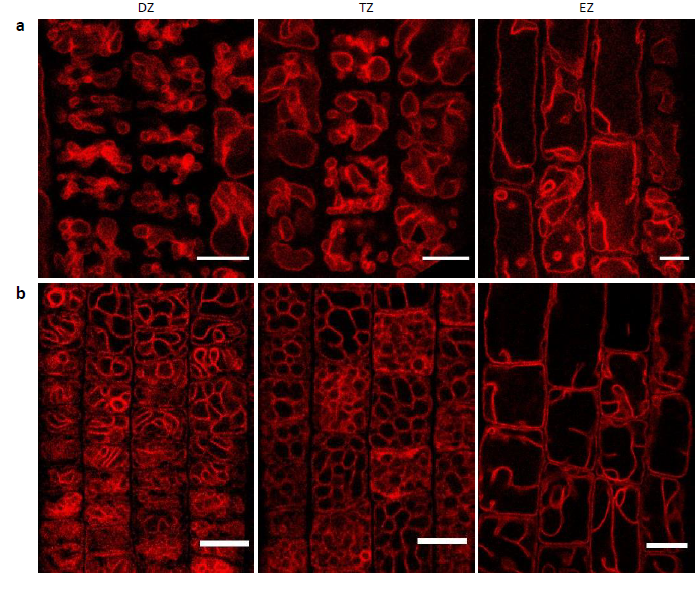


**Figure 7.** Vacuole comparison between Arabidopsis and rice root cells. (**a**) The vacuole morphology in Arabidopsis roots labelled with mCherry-VAMP711. (**b**) The vacuole morphology in rice root labelled with mCherry-OsTIP1;1. All images were from the epidermal cells. Scale bars: 10 µm.


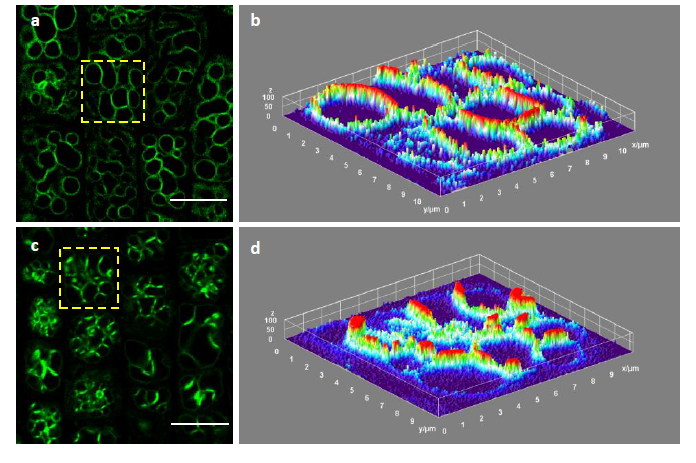


**Figure 8.** Vacuole response to salt treatment in rice roots. (**a**) The vacuole morphology in control rice root cells in the transition zone. (**b**) 3D surface plot of signal intensity as indicated by the yellow dashed rectangle in (**a**). (**c**) The vacuole morphology in rice root cells in the transition zone treated with 150 mM NaCl for 50 min. (**d**) 3D surface plot of signal intensity as indicated by the yellow dashed rectangle in (**c**). Scale bars: 10 µm.

| 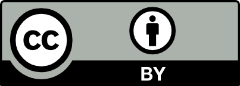 | © 2020 by the authors. Submitted for possible open access publication under the terms and conditions of the Creative Commons Attribution (CC BY) license (http://creativecommons.org/licenses/by/4.0/). |
| --- | --- |
